# Barriers to a Healthy Lifestyle Among Obese Patients Attending Primary Care Clinics in Al-Ahsa, Saudi Arabia

**DOI:** 10.7759/cureus.69036

**Published:** 2024-09-09

**Authors:** Ali Salah Almohammedsaleh, Yasin Yousef Alshawaf, Ahmed Mohammed Alqurayn

**Affiliations:** 1 Family and Community Medicine, Family Medicine Academy, Al-Hofuf, SAU

**Keywords:** barriers, health coach, healthy lifestyle, diet adherence, obesity

## Abstract

Obesity is a significant public health issue in Saudi Arabia, contributing to high morbidity and mortality rates. This study aimed to identify the barriers preventing obese patients from adhering to healthy diets and regular exercise while following a health coach in primary healthcare centers in Al-Ahsa. A cross-sectional study of 283 obese adults revealed that major barriers included lack of energy, willpower, and time for exercise, as well as lack of willpower, social influence, and time for maintaining a healthy diet. These barriers were significantly associated with demographic factors such as obesity grade, comorbidities, and educational level. Addressing these barriers is essential for developing effective interventions to support lifestyle changes in obese patients.

## Introduction

Obesity is a disorder characterized by a disproportionate increase in body weight to height, mainly due to the accumulation of fat, and is considered a pandemic of the present century by many international health institutions [1]. Many risk factors, including a sedentary lifestyle, a reduction of regular physical activity, and the consumption of unhealthful meals, can result in overweight and obesity., e.g., a high-carb diet and excessive sugar intake, as well as metabolic, endocrine, genetic, and environmental factors, are currently considered the main common causes for the obesity [2]. In addition, other contributing factors are the history of obesity in the family, the long duration of TV watching, and higher socioeconomic status [3]. Many noncommunicable illnesses and poor health status are linked with increasing body weight like type 2 diabetes, hypertension, cardiovascular diseases, stroke, osteoarthritis, gall stones, benign prostatic hyperplasia, obstructive sleep apnea, and some cancers like adenocarcinoma of the esophagus, gastric cardia, endometrial, breast, and colon cancers, leading to a massive impact on public health and related health economics [4]. Obesity is defined as body mass index (BMI) ≥30 kg/m^2^ and can be further stratified by class: class 1 (BMI = 30.0 to 34.9 kg/m^2^), class II (BMI = 35.0 to 39.9 kg/m^2^), and class III and above (BMI ≥ 40 kg/m^2^), and individuals with a BMI between 25 and 29.9 kg/m^2^ are considered overweight [5-6]. In 2016, more than 1.9 billion persons aged 18 and older (39% of the world population) were overweight, and almost 650 million of these people (13% of the population) were obese, which represented nearly threefold the number of obesities in 1975. In 2000, an unhealthy diet and decreased physical activity were linked to weight gain, contributing to 15% of deaths in the USA. Obesity reduces life expectancy by four to seven years [7]. By 2025, it is anticipated that 2.7 billion adults will be overweight, and more than one billion will be obese if current rates do not fall [2]. The Gulf countries (Saudi Arabia, Kuwait, United Arab Emirates, Bahrain, Qatar, and Oman) have the highest rate of obesity worldwide [8]. The World Health Organization's World Health Survey data for Saudi Arabia were updated by the Saudi Ministry of Health (SAWHS). According to this survey, 20% of people in KSA are obese, with greater rates among women (21%), those who live in cities (21%), and those with a low education level (29%). Moreover, obesity increases with age, rising from 10% in the age group of 18 to 29 to 20% in the 70 to 79 age group to 22% in the elderly above the age of 80 [9]. According to a recent study, the central area of Saudi Arabia has a greater prevalence of obesity among children and adolescents (21%) compared to the southwest and the northern regions [8]. Consequently, SA suffers a higher share of deaths attributable to obesity (18% vs. 8% globally) and a higher death rate by obesity (116.7 per 100,000 vs. 60 per 100,000 globally) [10]. Diet, physical exercise, and behavioral change are the three most important strategies for managing obesity because they all decrease the risk of becoming obese and improve both mental and physical health [11]. A healthy diet is important in protecting against non-communicable diseases. In Finland, the North Karelia project, community-based, showed that a healthy diet contributes to reducing the risk of coronary heart disease by 73% over 25 years [12]. These interventions can be easier achieved when a person receives health coach counseling and continues to follow up with him [13]. One study demonstrated that health coach counseling leads to significant weight loss [14]. Another study emphasizes that the use of a health coach is associated with weight loss through lifestyle interventions [15]. Also, research done in 2018 on pregnant women under 16 weeks of gestation found that the majority of participants (81%, 21 of 26) said that the health coach helped them reach their goals and kept them motivated [16]. However, there are many barriers to losing weight among patients attending primary care clinics and being seen by a health coach. Three main limitations are lack of training, limited consultation time, and lack of the necessary motivation to lose weight for the patients [11]. Thirty-five publications were done in North America that showed the motivational factors and challenges to weight loss, and most of the participants were female. Most of these published studies shared the same three levels of barriers and motivations. include individual, environmental, and interventional levels. Regarding the personal level, attitudes, health concerns, and physical modifications were the main barriers. At the environmental level, social accountability, social support, and changeable and unchangeable aspects of the community were the main barriers. Finally, barriers at the intervention level involved delivery, design, and content. Recently, it was found that busy lifestyles and a lack of restaurants with healthy food choices have led the younger age group to consume fast food [17]. Research conducted in the UK revealed that the capacity to maintain long-term weight reduction successfully was limited by adverse weather conditions, natural occurrences including accidents, illnesses, injuries, work responsibilities, poor time management, and inability to resist the need to eat [4]. Lack of a safe environment was found as the main identified barrier to physical activity like walking, biking, and playing [18]. In the UK, a study showed extreme weather conditions and natural phenomena, such as accidents, injuries, ill health, work commitments, poor time management, and resisting the temptation for food, constrained successful long-term weight loss maintenance [19]. Regarding Saudi Arabia, a study was conducted in 2009 at the College of Medicine, King Saud University, Riyadh, Saudi Arabia. It included 450 participants ranging from 15 to 80 years and showed that 82.4% of Saudis were physically inactive. Women were more physically inactive (87.6%) compared to men (71.5%). Corresponding to that, younger age groups showed a considerable increase in physical inactivity. Lack of willpower was the most frequent obstacle to a healthy diet (80.3%). The middle-aged group (30-45 years) and those who had never been married are the most frequent group mentioned with lack levels of willpower. A healthy diet is difficult to maintain in the study group (72.4%) due to a lack of social support. It was much greater among individuals with less education than a university degree and in middle-aged people (30-45 years old) [12]. Another study that was conducted in the Al-Qassim region in 2017 showed that the main barriers to losing weight were lack of family support and unhealthy eating during social gatherings.

Obesity is a global health problem that leads to a high rate of mortality and morbidity. Saudi Arabia has one of the highest rates of overweight and obesity among developed countries. According to our reviews, only a few research were conducted in Saudi Arabia talking about barriers that prevent obese patients from losing weight and how they can overcome these obstacles; our aim of the study is to identify these barriers among obese patients who are following health coach in primary health care centers in Al-Ahsa.

## Materials and methods

Study design

A descriptive cross-sectional study in primary healthcare centers in Al-Ahsa, Saudi Arabia, was conducted.

Study population

In this cross-sectional study, obese participants aged ≥ 18 and their BMI ≥ 30 and < 40 were used as a study group. The participants visited the health coach at least once.

Exclusion criteria

The exclusion criteria included obesity secondary to genetic syndromes, hypothalamic or hormonal alterations, any liver, heart, or kidney diseases causing edema, which could affect body weight and/or waist circumference, terminal illness, cognitive impairment preventing the collection of information, and pregnancy or breastfeeding.

Study area

The study was conducted in primary health care centers in four public health sectors: North, East, Middle, and South sectors, in Al-Ahsa, Saudi Arabia.

Sampling and sample size

A convenient sampling technique was used to acquire available participants, and the sample size (320 participants) was calculated using a statistical calculator with a 5% margin of error and a 95% confidence level, using the website https://www.surveysystem.com/sscalc.htm.

Data collection tool

Data were collected through a self-administered questionnaire containing three major parts. The first part is about sociodemographic variables (age, marital status, occupation, education, and economic status), and the second and third parts cover questions about barriers to physical activity and a healthy diet. The questionnaire was distributed among obese participants who visited health coach clinics in primary health care. It is validated from the Centers for Disease Control and Prevention (CDC) website [20]. We ensured to stimulate them to respond to this research by communicating with them on their private contact numbers, and it was distributed through social media. It was formed in Arabic to facilitate the response by participants in Al-Ahsa (Supplementary Material 1).

Data analysis

After data were extracted, it was revised, coded, and fed to statistical software IBM SPSS version 22 (IBM Corp., Armonk, NY). All statistical analysis was done using two-tailed tests. A p-value of less than 0.05 was statistically significant. As for barriers to adhering to physical activities, the seven categories of main barriers to physical activity (lack of time, lack of social influence, lack of energy, lack of resources, lack of willpower, fear of injury, and lack of skill), and five categories for health diet barriers (lack of social influence, lack of time, lack of knowledge, low socioeconomic state, and lack of willpower) were assessed by summing up the discrete items score for each barrier category (three questions for each) with a total score ranged from 0 to nine for each barrier category. The barrier category with a total score of five or more was considered an important barrier for the answered cases. Descriptive analysis based on frequency and percent distribution was done for all variables participants' bio-demographic data and reported barriers against adhering to a healthy diet and exercise. Also, participants' frequency of visiting health educators and the reported barriers and barriers categories were graphed. Cross tabulation was used to assess factors associated with participants visiting health educators and to assess the distribution of barriers categories by obese cases bio-demographic data using Person's chi-square test and exact probability test for small frequency distributions.

## Results

Out of 320 participants, 283 eligible obese patients completed the study questionnaire. Patient ages ranged from 18 to over 60 years, with a mean age of 42.6 ± 11.9 years old. Exactly 197 (69.6%) of the participants were females, 208 (73.5%) were married, and 112 (39.6%) had a university level of education or higher, while 74 (26.1%) had an education below the secondary level. As for employment, 185 (65.4%) were employed, and 185 (65.4%) had a monthly income of less than 5,000 Saudi Arabian Riyal (SAR), while 56 (19.8%) had a monthly income exceeding 10,000 SAR. A total of 140 (49.5%) were classified as grade I obese patients, and 143 (50.5%) as grade II obese. Considering comorbidities, 97 (34.3%) were diabetic, 82 (29%) were hypertensive, and 58 (20.5%) had osteoarthritis, while 119 (42%) had no comorbidities (Table 1).

**Table 1:** Bio-demographic characteristics of study obese patients attending primary healthcare centers, Al-Ahsa, Saudi Arabia (n = 283)

Bio-demographic data	No	%
Age in years		
18-25	26	9.2%
25-40	110	38.9%
40-60	140	49.5%
>60	7	2.5%
Gender		
Male	86	30.4%
Female	197	69.6%
Marital status		
Single	75	26.5%
Married	208	73.5%
Educational level		
Below secondary	74	26.1%
Secondary	97	34.3%
University/more	112	39.6%
Employment		
Unemployed	98	34.6%
Employed	185	65.4%
Income		
<5,000 SAR	185	65.4%
5,000-10,000 SAR	42	14.8%
10,000-15,000 SAR	33	11.7%
>15,000 SAR	23	8.1%
Obesity		
Obesity grade I	140	49.5%
Obesity grade II	143	50.5%
Comorbidities		
Diabetes mellitus	97	34.3%
Hypertension	82	29.0%
Osteoarthritis	58	20.5%
Hypothyroidism	17	6.0%
None	119	42.0%

**Figure 1:**
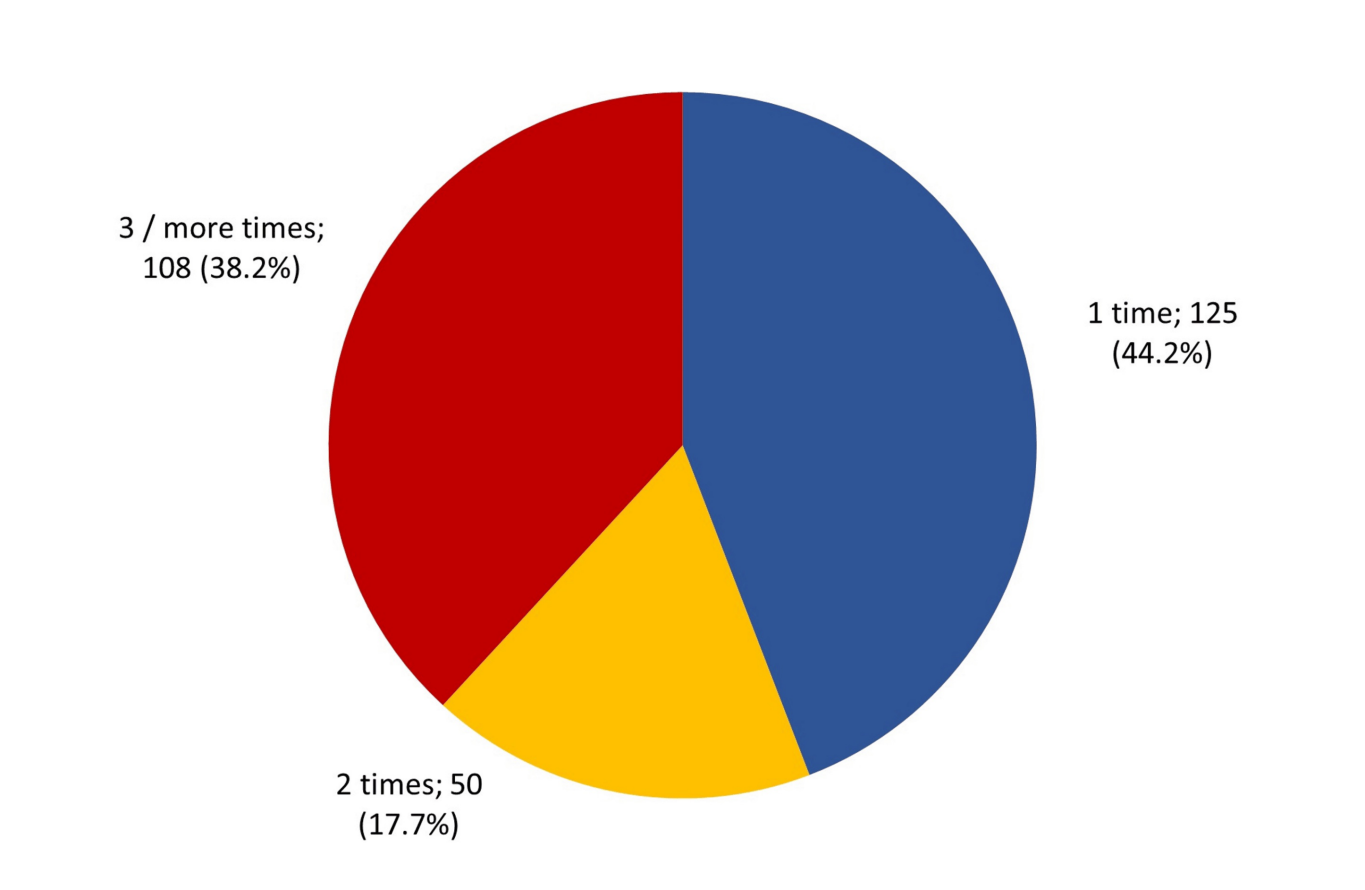
Frequency of visiting health educators in the primary health care centers by obese participants, Al-Ahsa, Saudi Arabia

A total of 125 (44.2%) of the obese patients visited the health educator only once, 50 (17.7%) visited two times, and 108 (38.2%) visited three times or more (Figure 1).

**Figure 2:**
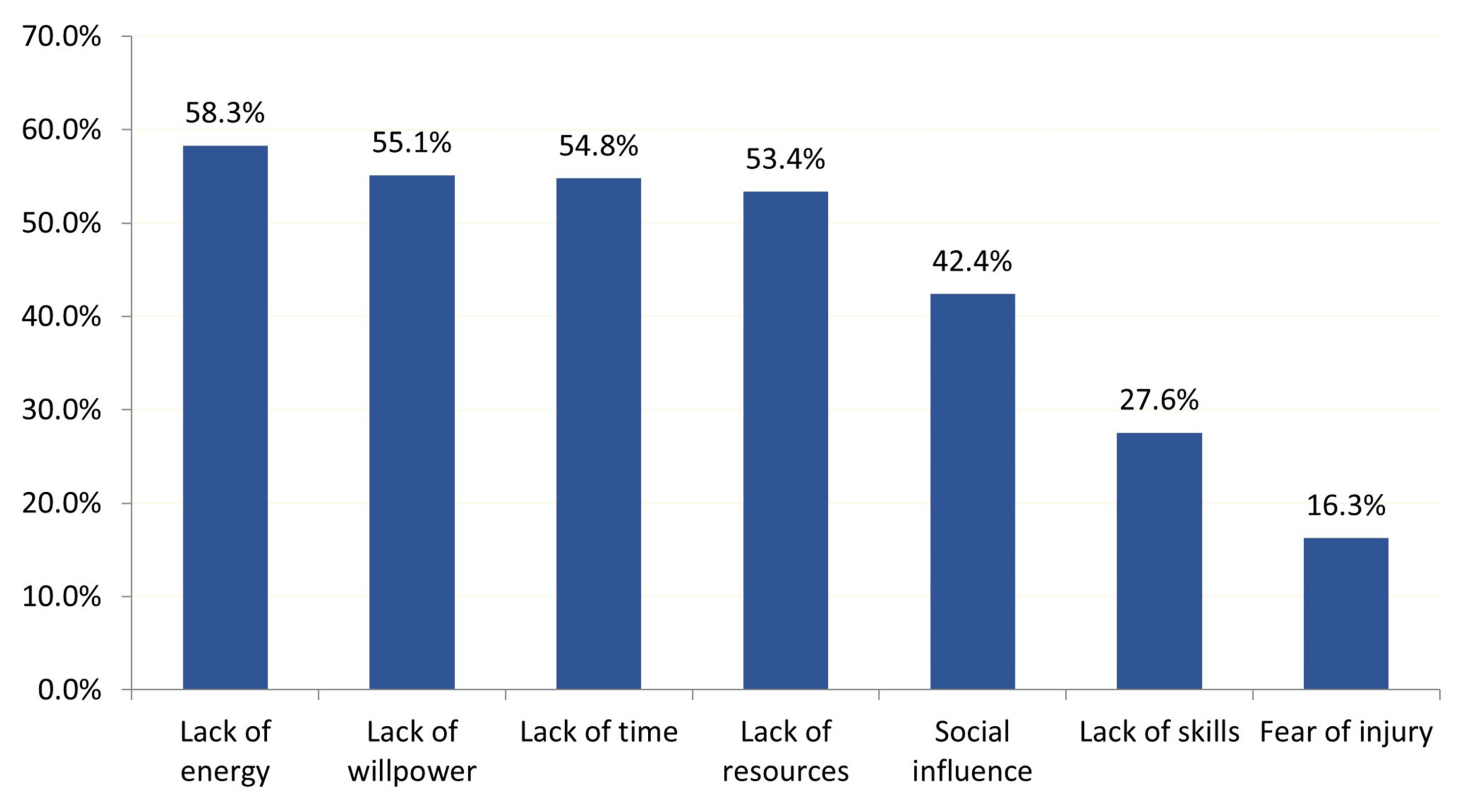
Barriers categories that prevent obese patients from adhering to regular exercises, Al-Ahsa, Saudi Arabia

As for barriers categories that prevent obese patients from adhering to regular exercises, the most reported included lack of energy 183 (58.3%), lack of willpower 176 (55.1%), lack of time 175 (54.8%), lack of resources 170 (53.4%), social influence 135 (42.4%), lack of skills 88 (27.6%), and fear of injury was the least reported barriers 52 (16.3%) (Figure 2).

**Figure 3:**
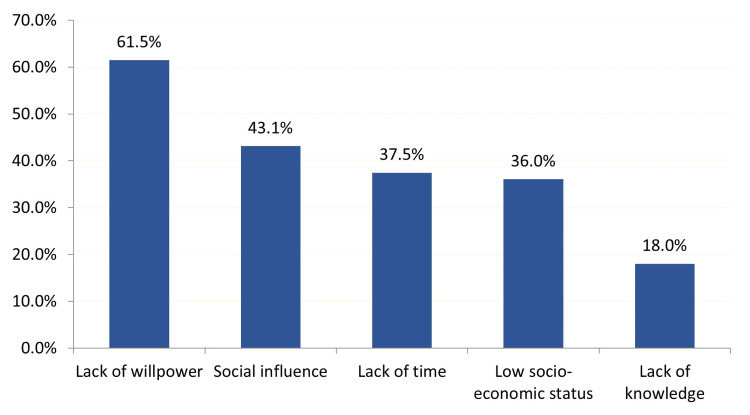
Barriers categories that prevent obese patients from adhering to a healthy diet, Al-Ahsa, Saudi Arabia

About barriers categories that prevent obese patients from adhering to a healthy diet, the most reported were lack of willpower 196 (61.5%), social influence 137 (43.1%), lack of time 120 (37.5%), low socioeconomic status 115 (36%), and lack of knowledge 57 (18%) (Figure 3).

Considering lack of time, it was significantly more reported among obese cases with no comorbidities than others (74 (62.7%) vs. 81 (49.1%), respectively; p = 0.023). Considering the social influence barrier, it was significantly higher among grade II obese cases than grade I obesity (72 (50.3%) vs. 48 (34.3%), respectively; p = 0.006) and among 69 (55.2%) of those who visited health educator only once vs. 35 (32.4%) of others who did for three times or more (p = 0.001). Also, 95 (66.4%) of grade II obese cases reported lack of energy as a barrier to exercising compared to 70 (50%) of grade I obese (p = 0.005) and 77 (65.3%) of those with no comorbidities reported for lack of energy in comparison to 88 (53.3%) of others (p = 0.045). Additionally, lack of willpower was reported among 73 (61.9%) of obese cases with no comorbidities vs. 83 (50.3%) of others with comorbidities (p = 0.049). Exactly three (42.9%) of old-age obese cases reported fear of injury as an important barrier (p = 0.012), as did 39 (18.8%) married patients vs. seven (9.3%); p = 0.049), 21 (28.4%) low educated obese cases (p = 0.003), and 36 (21.8%) of those with comorbidities vs. 10 (8.5%) (p = 0.003). Lack of skills as an important barrier was reported by four (57.1%) of old-age obese patients compared to five (19.2%) of young-age patients (p = 0.022), and by 64 (30.8%) married cases compared to 14 (18.7%) (p = 0.044). It was also reported by 29 (39.2%) patients with low education levels compared to 21 (18.8%) with higher education (p = 0.009). The same barrier was also reported among 47 (32.9%) grade II obese patients compared to 31 (22.1%) (p = 0.044) grade I obese patients and among 54 (32.7%) patients with comorbidities vs. 24 (20.3%) without (p = 0.021). Exactly 62 (63.3%) of unemployed obese cases reported lack of resources as an important barrier compared to 89 (48.1%) of employed cases (p = 0.015) and also was reported by 30 (71.4%) of low-income cases (5,000-10,000 SAR) vs. eight (34.8%) of others with monthly income exceeding 15,000 SAR (p = 0.031). Additionally, 89 (62.2%) of grade II obesity cases reported a lack of resources as a barrier to exercising in comparison to 62 (44.3%) of grade I obese cases (p = 0.002) (Table 2).

**Table 2:** Distribution of barriers to adhering to exercises by obese participants bio-demographic data

Factors	Lack of time		Social influence		Lack of energy		Lack of willpower		Fear of injury		Lack of skills		Lack of resources	
	No	%	No	%	No	%	No	%	No	%	No	%	No	%
Age in years														
18-25	17	65.4%	13	50.0%	16	61.5%	20	76.9%	2	7.7%	5	19.2%	17	65.4%
25-40	67	60.9%	50	45.5%	71	64.5%	59	53.6%	11	10.0%	22	20.0%	63	57.3%
40-60	67	47.9%	54	38.6%	75	53.6%	75	53.6%	30	21.4%	47	33.6%	67	47.9%
>60	4	57.1%	3	42.9%	3	42.9%	2	28.6%	3	42.9%	4	57.1%	4	57.1%
p-value	0.134		0.599		0.278		0.065		0.012*		0.022*		0.269	
Gender														
Male	44	51.2%	33	38.4%	45	52.3%	45	52.3%	13	15.1%	23	26.7%	45	52.3%
Female	111	56.3%	87	44.2%	120	60.9%	111	56.3%	33	16.8%	55	27.9%	106	53.8%
p-value	0.420		0.365		0.178		0.532		0.732		0.839		0.818	
Marital status														
Single	37	49.3%	33	44.0%	42	56.0%	38	50.7%	7	9.3%	14	18.7%	43	57.3%
Married	118	56.7%	87	41.8%	123	59.1%	118	56.7%	39	18.8%	64	30.8%	108	51.9%
p-value	0.270		0.744		0.637		0.365		0.049*		0.044*		0.421	
Educational level														
Below secondary	40	54.1%	31	41.9%	39	52.7%	35	47.3%	21	28.4%	29	39.2%	42	56.8%
Secondary	47	48.5%	41	42.3%	52	53.6%	51	52.6%	14	14.4%	28	28.9%	46	47.4%
University/more	68	60.7%	48	42.9%	74	66.1%	70	62.5%	11	9.8%	21	18.8%	63	56.3%
p-value	0.204		0.991		0.100		0.103		0.003*		0.009*		0.351	
Employment														
Unemployed	58	59.2%	41	41.8%	63	64.3%	59	60.2%	13	13.3%	26	26.5%	62	63.3%
Employed	97	52.4%	79	42.7%	102	55.1%	97	52.4%	33	17.8%	52	28.1%	89	48.1%
p-value	0.278		0.888		0.137		0.211		0.321		0.778		0.015*	
Income														
<5,000 SAR	104	56.2%	85	45.9%	109	58.9%	101	54.6%	33	17.8%	55	29.7%	96	51.9%
5,000-10,000 SAR	22	52.4%	14	33.3%	25	59.5%	24	57.1%	8	19.0%	11	26.2%	30	71.4%
10,000-15,000 SAR	17	51.5%	13	39.4%	17	51.5%	16	48.5%	3	9.1%	8	24.2%	17	51.5%
>15,000 SAR	12	52.2%	8	34.8%	14	60.9%	15	65.2%	2	8.7%	4	17.4%	8	34.8%
p-value	0.928		0.386		0.863		0.654		0.425		0.604		0.031*	
Obesity														
Obesity grade I	72	51.4%	48	34.3%	70	50.0%	71	50.7%	18	12.9%	31	22.1%	62	44.3%
Obesity grade II	83	58.0%	72	50.3%	95	66.4%	85	59.4%	28	19.6%	47	32.9%	89	62.2%
p-value	0.264		0.006*		0.005*		0.140		0.125		0.044*		0.002*	
Comorbidities														
Yes	81	49.1%	64	38.8%	88	53.3%	83	50.3%	36	21.8%	54	32.7%	83	50.3%
No	74	62.7%	56	47.5%	77	65.3%	73	61.9%	10	8.5%	24	20.3%	68	57.6%
p-value	0.023*		0.146		0.045*		0.049*		0.003*		0.021*		0.223	
Number of visits to health educator														
One time	66	52.8%	69	55.2%	74	59.2%	73	58.4%	19	15.2%	36	28.8%	70	56.0%
Two times	32	64.0%	16	32.0%	28	56.0%	30	60.0%	7	14.0%	16	32.0%	29	58.0%
Three/more times	57	52.8%	35	32.4%	63	58.3%	53	49.1%	20	18.5%	26	24.1%	52	48.1%
p-value	0.352		0.001*		0.928		0.270		0.706		0.536		0.375	

Considering lack of willpower, it was significantly higher among cases with comorbidities than among those without (11 (66.7%) vs. 64 (54.2%); p = 0.034). Lack of knowledge was reported by 21 (28.4%) of obese patients with low education level compared to 15 (13.4%) with high education (p = 0.024), and by 40 (24.2%) patients with comorbidities compared to 11 (9.3%) without comorbidities (p = 0.001). A total of 64 (44.8%) grade II obese cases reported a lack of time as a barrier compared to 42 (30%) of grade I obese cases (p = 0.010). Social influence was reported by 17 (65.4%) of young-age obese cases compared to two (28.6%) of old-age cases (p = 0.049). A total of 34 (45.3%) of single cases reported low socioeconomic status as an important barrier to a healthy diet vs. 68 (32.7%) of married cases (p = 0.049) (Table 3).

**Table 3:** Distribution of barriers to adhering to a healthy diet by obese participant's bio-demographic data

Factors	Lack of willpower		Lack of knowledge		Lack of time		Social influence		Low socioeconomic status	
	No	%	No	%	No	%	No	%	No	%
Age in years										
18-25	15	57.7%	2	7.7%	10	38.5%	17	65.4%	14	53.8%
25-40	73	66.4%	15	13.6%	46	41.8%	47	42.7%	41	37.3%
40-60	83	59.3%	32	22.9%	47	33.6%	56	40.0%	46	32.9%
>60	3	42.9%	2	28.6%	3	42.9%	2	28.6%	1	14.3%
p-value	0.462		0.109		0.595		0.049*		0.127	
Gender										
Male	56	65.1%	17	19.8%	33	38.4%	35	40.7%	32	37.2%
Female	118	59.9%	34	17.3%	73	37.1%	87	44.2%	70	35.5%
p-value	0.407		0.614		0.833		0.588		0.787	
Marital status										
Single	41	54.7%	11	14.7%	32	42.7%	37	49.3%	34	45.3%
Married	133	63.9%	40	19.2%	74	35.6%	85	40.9%	68	32.7%
p-value	0.157		0.378		0.277		0.204		0.049*	
Educational level										
Below secondary	44	59.5%	21	28.4%	32	43.2%	27	36.5%	23	31.1%
Secondary	59	60.8%	15	15.5%	33	34.0%	45	46.4%	38	39.2%
University/more	71	63.4%	15	13.4%	41	36.6%	50	44.6%	41	36.6%
p-value	0.853		0.024*		0.454		0.395		0.544	
Employment										
Unemployed	57	58.2%	15	15.3%	43	43.9%	35	35.7%	38	38.8%
Employed	117	63.2%	36	19.5%	63	34.1%	87	47.0%	64	34.6%
p-value	0.403		0.387		0.104		0.067		0.486	
Income										
<5,000 SAR	110	59.5%	31	16.8%	69	37.3%	84	45.4%	67	36.2%
5,000-10,000 SAR	32	76.2%	8	19.0%	22	52.4%	18	42.9%	18	42.9%
10,000-15,000 SAR	18	54.5%	5	15.2%	9	27.3%	11	33.3%	8	24.2%
>15,000 SAR	14	60.9%	7	30.4%	6	26.1%	9	39.1%	9	39.1%
p-value	0.185		0.421		0.081		0.608		0.401	
Obesity										
Obesity grade I	82	58.6%	23	16.4%	42	30.0%	61	43.6%	48	34.3%
Obesity grade II	92	64.3%	28	19.6%	64	44.8%	61	42.7%	54	37.8%
p-value	0.319		0.490		0.010*		0.877		0.543	
Comorbidities										
Yes	110	66.7%	40	24.2%	60	36.4%	68	41.2%	54	32.7%
No	64	54.2%	11	9.3%	46	39.0%	54	45.8%	48	40.7%
p-value	0.034*		0.001*		0.654		0.446		0.170	
Number of visits to health educator										
One time	80	64.0%	17	13.6%	44	35.2%	49	39.2%	49	39.2%
Two times	34	68.0%	12	24.0%	24	48.0%	27	54.0%	18	36.0%
Three/more times	60	55.6%	22	20.4%	38	35.2%	46	42.6%	35	32.4%
p-value	0.243		0.195		0.237		0.201		0.560	

## Discussion

A total of 283 participants were included in this study to identify the barriers to a healthy lifestyle among obese patients attending primary care clinics in the Al-Ahsa governorate. There were 37 participants excluded, as they did not meet the criteria of this study. Most of the participants, 125 (44.2%), of this study visited the healthcare educator only once compared to 50 (17.7%) and 108 (38.2%) of the participants who visited twice and three times or more, respectively.

Regarding physical activity, age differences, marital status, and level of education showed two significant differences (p < 0.05) with fear of injury and lack of skills. The age difference can be justifiable as older participants (>60 years old) will fear getting injured and will have lower skills compared to younger participants. A similar study done earlier in Riyadh showed that the age differences resulted in only one significant difference among one barrier, which is fear of injury, which supports our finding [12]. Also, marital status showed only a significant difference with fear of injury, while level of education showed two significant differences with lack of skills and fear of injury in the Riyadh study [12]. This could be explained as married participants have more responsibilities with their families, and for that reason, they fear injury, and they will have less time to increase their skill levels. Lower participant levels showed that they were more prone to fear and had low skill levels. Gender differences did not show any significant differences (p > 0.05). In contrast, a Mexican study was conducted to evaluate different barriers that affect adherence to a healthy diet and physical activity and found that obese female participants had more barriers than others [20]. An earlier study found that male participants had higher engagement in vigorous activity than females, while females had higher engagement in moderate activity [21]. Employment type and income showed significant differences, with the only barrier being a lack of resources, whereas unemployed and low-income participants showed higher percentages. This could be because they do not have enough money to spend on buying exercise equipment or paying for gym subscription fees. A North Carolina study supported this finding, as about half of the participants (52%) reported that places that are not affordable for exercise are a barrier to exercise [22].

For a healthy diet, a significant correlation (p < 0.05) was found between age differences and social influence. In comparison, the Riyadh study showed substantial differences among different barriers, such as lack of willpower, lack of social support, and lack of time. Similarly, our study and Riyadh’s study did not show any significant difference between gender and monthly income with the different barriers [12]. Marital status showed one significant difference with low socioeconomic status, as single participants showed a higher percentage than married participants. This could be because married participants, especially females, got financial help from their husbands, as males are the main providers in the family. Lack of knowledge was the only barrier to showing a significant difference (p = 0.024) in the education level of participants. Higher education level (university degree) showed better knowledge than lower education level (below secondary level).

Lack of energy was ranked as the top barrier that prevents obese patients from adhering to regular exercise (134 (58.3%)). This is not surprising as lack of energy was among the top three barriers to exercise (49%), as reported by a Colombian study that was aimed to measure the barriers to physical activity in a population of general adults [23]. Obese patients tend not to adhere to physical activity, and for that reason, lack of energy was the top barrier in this study. Moreover, lack of time was the third top barrier to adherence in our study (54.8%). Lack of time was the top barrier in a Kuwait study that examined the cultural factors that affect adherence to exercise [24]. Most of the participants in the current study were employed (185(65.4%)), and 208 (73.5%) of the participants were married, which made them busy with work and family.

Our study showed that lack of willpower is the top barrier that prevents obese patients from adhering to a healthy diet with 126 (55.1%). A study conducted in primary healthcare in Oman to evaluate the different barriers to leisure time physical activity in a publication on type 2 diabetes found that lack of willpower is the top barrier with 44% [25]. It might be difficult for the participants to eat a healthy diet, especially as they want freedom in their food choices. A Spanish study found similar results: lack of willpower was the top barrier to adhering to healthy food [26].

Different barriers were examined to find if they affect the frequency of visits to health educators for obese patients. Only the lack of social influence barrier was found to be statistically significant (p = 0.001) as a barrier to exercise. The frequency of visiting health educators is mostly one-time compared to other choices, which suggests that social influence can increase the frequency of visiting health educators. On the other hand, no barrier was found to be statistically significant with a healthy diet. The examined factors were not associated with any significant difference with a healthy diet in the sample used, so other factors should be examined in the future. 

In addition, the participants with comorbidity (diabetes mellitus, hypertension, osteoarthritis, and hypothyroidism) showed to visit the health educator three or more times compared to the participants who did not have any comorbidity. The participants with comorbidity might be more concerned about their health status, and for that reason, they visit health educators more often compared to the participants who do not have any comorbidity.

This study showed the lack of physical activity and healthy food by obese patients in Al-Ahsa governorate. For that reason, awareness of the importance of doing physical exercises and eating healthy food is needed via seminars, pamphlets, and social media. In addition, the health coaches need to improve their programs by, for instance, engaging different people together, which could enhance their social influence. This could help in increasing their adherence to physical activity and a healthy diet.

The study on barriers to a healthy lifestyle among obese patients attending primary care clinics in Al-Ahsa has several limitations. The use of a convenient sampling method and a relatively small sample size may limit the generalizability of the findings, as the sample may not fully represent the diverse population. The reliance on self-reported data through questionnaires could introduce bias, and the cross-sectional design limits the ability to establish causality between identified barriers and lifestyle adherence. Additionally, the study's focus on a single city and the absence of longitudinal data restrict the understanding of regional variations and long-term effects. Furthermore, the study may not capture all potential barriers, particularly those that are culturally specific or less commonly discussed, necessitating caution in interpreting the results and suggesting the need for further research.

## Conclusions

In conclusion, lack of energy is the top barrier that prevents obese patients from adhering to regular exercise, while lack of willpower is the top barrier that prevents obese patients from adhering to a healthy diet among those attending primary care clinics in Al-Ahsa governorate. More educational courses are needed to motivate and increase the awareness of obese patients about physical exercise and healthy food.

## Appendices

**Table 4:** Arabic questionnaire

نعم- غالبا (3)	نعم- أحيانا (2)	لا- نادرا (1)	لا- أبدا (0)	
				1- يومي مشغول جدا لذلك لا أستطيع أن أجعل لي وقتا خاص للتمارين ضمن جدولي اليومي
				2- لا أحد من أفراد عائلتي أو أصدقائي يفضل ممارسة الرياضة لذلك فإنني لا أجد فرصه للممارسة
				3- أكون متعب جدا بعد العمل فلا أستطيع ممارسة الرياضة
				4- كنت أفكر في زيادة التمارين الرياضية لكن لا يبدو أنني أستطيع البدء في ذلك
				5- أنني كبير في السن لذا فممارسة الرياضة تشكل خطرا علي
				6- لا أمارس الرياضة كثيرا وذلك لأنني لم أتعلم مهارات أي نوع من أنواع الرياضة
				7- (المسابح, المسارات, الدراجات,...الخ) غير متوفرة للاستخدام بالنسبة لي
				8- ممارسة الرياضة تأخذ الكثير من وقت التزامات أخرى مثل وقت العمل والأسرة
				9- أنا أشعر بالخجل من منظري وأنا أمارس الرياضة مع الأخرين
				10- أنا لا أحصل على القدر الكافي من النوم فلا أستطيع الاستيقاظ مبكرا أو البقاء في الليل لممارسة الرياضة
				11- من الأسهل علي إيجاد الأعذار لعدم ممارسة الرياضة
				12- أنا اعرف ناسا كثيرين آذوا أنفسهم بممارسة من جراء كثرة ممارسة الرياضة
				13- أنا أرى انه لا يمكن تعلم أي رياضة جديدة في مثل سني
				14- ممارسة الرياضة أمر مكلف جدا فيجب علي شراء أدوات رياضية أو أخذ دروس رياضية
				15- وقت فراغي أثناء اليوم ضيق جدا لا يكفي أداء التمارين الرياضية
				16- نشاطاتي الاجتماعية المعتادة مع العائلة والأصدقاء لا تتضمن أي نشاط رياضي
				17- أنا متعب\ه جدا خلال أيام الأسبوع، وأحتاج أيام العطلة للراحة وليس الرياضة
				18- أريد أمارس المزيد، ولكن يبدو أني لا أستطيع أن أستمر على شيء معين
				19- أنا أخاف أن أؤذي نفسي أو أصاب بأزمة قلبية
				20- ليس لدي القدرة الكافية لتحويل ممارسة الرياضة الى متعة
				21- لو كانت هناك أجهزة رياضية وغرف مخصصة للاستحمام في موقع العمل لكان من الأرجح أقوم بممارسة التمارين الرياضية
